# Clinical outcomes of HIF-2**α** inhibition in Von Hippel–Lindau disease: a multicenter real-world study

**DOI:** 10.1093/oncolo/oyag331

**Published:** 2026-08-18

**Authors:** Mousab Al Abbas, Jabra Zarka, Alvin C Silva, Filippo Cingolani, Muhammed Uzair Sarfraz, Albert Jang, Muhammad Ali Khan, Fouad Nahhat, Winston Tan, Brian A Costello, Haidar Abdul-Muhsin, Irbaz Bin Riaz, Yousef Zakharia

**Affiliations:** Division of Hematology and Oncology, Department of Medicine, Mayo Clinic Arizona, Phoenix, AZ 85054, United States; Division of Internal Medicine, University of South Alabama, Mobile, AL 36617, United States; Division of Hematology and Oncology, Department of Medicine, Mayo Clinic Rochester, Rochester, MN 55905, United States; Division of Radiology, Mayo Clinic Arizona, Phoenix, AZ 85054, United States; Division of Hematology and Oncology, Department of Medicine, Mayo Clinic Arizona, Phoenix, AZ 85054, United States; Division of Hematology and Oncology, Department of Medicine, Mayo Clinic Arizona, Phoenix, AZ 85054, United States; Division of Hematology and Oncology, Department of Medicine, Mayo Clinic Rochester, Rochester, MN 55905, United States; Division of Hematology and Oncology, Department of Medicine, Mayo Clinic Arizona, Phoenix, AZ 85054, United States; Division of Hematology and Oncology, Department of Medicine, Mayo Clinic Arizona, Phoenix, AZ 85054, United States; Division of Hematology and Oncology, Department of Medicine, Mayo Clinic Florida, Jacksonville, FL 32224, United States; Division of Hematology and Oncology, Department of Medicine, Mayo Clinic Rochester, Rochester, MN 55905, United States; Division of Urology, Mayo Clinic Arizona, Phoenix, AZ 85054, United States; Division of Hematology and Oncology, Department of Medicine, Mayo Clinic Arizona, Phoenix, AZ 85054, United States; Division of Hematology and Oncology, Department of Medicine, Mayo Clinic Arizona, Phoenix, AZ 85054, United States

**Keywords:** belzutifan, VHL disease, real-world, HIF-2α

## Abstract

**Background and Objective:**

Management of von Hippel–Lindau (VHL) disease is challenging due to multisystem involvement and limited systemic treatment options. Belzutifan has shown promising efficacy in clinical trials; however, real-world data remain limited. We aimed to evaluate its effectiveness and safety in a real-world cohort.

**Methods:**

We conducted a retrospective study of 59 patients with VHL disease treated with belzutifan between August 2021 and January 2026. Objective response rates (ORR) for renal cell carcinoma (RCC), central nervous system hemangioblastomas (CNS HB), and pancreatic neuroendocrine tumors (pNET) were assessed by an independent radiologist. Retinal hemangioblastoma outcomes were evaluated through ophthalmology records by chart review.

**Results:**

Median follow-up was 29 months. ORR was 27.6%, 39.6%, and 85.7% for RCC, CNS HB, and pNET, respectively. Among 13 patients with retinal hemangioblastomas (20 eyes), 17 eyes (85%) showed improvement. At data cutoff, 47 patients (79.6%) remained on treatment, while 12 (20.3%) discontinued (7 due to adverse events, 3 to disease progression, and 2 to other reasons). The most common adverse event was anemia (86.4%). Grade ≥3 adverse events occurred in 10 patients (16.9%). The procedure rate per patient-year decreased from 0.35 in the 3 years prior to treatment to 0.08 during follow-up (Incidence Rate Ratio, 0.23; 95% CI, 0.12-0.47; *P *< .001)

**Conclusions:**

Belzutifan demonstrated efficacy across VHL-associated lesions, with a favorable safety profile. Most adverse events were grade 1-2. A reduction in procedure rate was observed after treatment initiation.

Implications for Practice This real-world study supports the use of belzutifan as an effective systemic treatment for patients with VHL disease, demonstrating durable activity across multiple VHL-associated tumors and a manageable safety profile. The observed reduction in procedure rate suggests that belzutifan may decrease the need for repeated surgical or local interventions, potentially reducing procedural burden while preserving disease control.

## Introduction

Von Hippel–Lindau (VHL) disease is an autosomal dominant disorder caused by germline mutations in the *VHL* gene, with an estimated incidence of approximately 1 in 27,300 live births.^1^ It is characterized by the development of multiple benign and malignant tumors across various organs, most commonly affecting the pancreas, central nervous system, retina, and kidneys, resulting in substantial morbidity.^2^ Patients frequently require repeated surgical or interventional procedures, contributing to significant lifetime procedural burden.^3^

VHL disease results from germline loss-of-function mutations in the *VHL* tumor suppressor gene.^4^ The *VHL* gene, located on chromosome 3p25, encodes the *VHL* protein (pVHL), which under normoxic conditions targets hypoxia-inducible factor-alpha (HIF-α) subunits for proteasomal degradation.^5^ Loss of functional *pVHL* leads to accumulation of *HIF-α*, promoting transcription of genes involved in angiogenesis and proliferation, including *VEGFA, PDGFB*, and *CCND1* a process primarily driven by *HIF-2α.*^5^ This dysregulated *HIF* signaling underlies the development of VHL-associated tumors.^6^

Given the central role of *HIF-2α* in *VHL* pathogenesis, its inhibition has emerged as a therapeutic strategy.^5^ Belzutifan (MK-6482), a selective *HIF-2α* inhibitor, has demonstrated efficacy in *VHL*-associated tumors and sporadic renal cell carcinoma (RCC) in clinical trials.^7^^,^^8^ However, real-world data on its effectiveness and safety in VHL disease remain limited. Therefore, we aimed to evaluate these outcomes in a real-world cohort.

## Methods

### Patients’ selection

A retrospective chart review was conducted of patients treated with belzutifan between August 2021 and January 2026 at Mayo Clinic Comprehensive Cancer Centers across three sites (Minnesota, Arizona, and Florida). Patients were identified through pharmacy records and included if they met one of the following criteria: a pathogenic germline *VHL* mutation with ≥1 VHL manifestation; a first-degree relative with VHL and ≥1 manifestation; or ≥2 VHL manifestations, with one of them being hemangioblastoma.^9^

Patients were required to have baseline CT or MRI within 3 months prior to treatment initiation, evaluable renal, pancreatic, and CNS disease, and at least one follow-up imaging study. Exclusion criteria included sporadic RCC, hemangioblastoma, paraganglioma, pheochromocytoma, SDHB-associated pheochromocytoma/paraganglioma, or missing baseline or follow-up imaging. This study was approved by the Institutional Review Board at Mayo Clinic (IRB No. 23-010859).

### Data collection

Data were collected from electronic medical records and included patient demographics, date of VHL disease diagnosis, initiation date of belzutifan, tumor reduction procedures with their dates, adverse events, dose reductions, treatment interruptions, and treatment discontinuation. Adverse events were extracted from clinical notes and graded according to Common Terminology Criteria for Adverse Events (CTCAE) v5. The tumor reduction procedures extracted are listed in Table S1. Data abstraction was conducted using a standardized tool by two independent reviewers, with discrepancies resolved by consensus.

### Study endpoints

The primary endpoint was objective response rate (ORR), defined as partial response (PR) or complete response (CR) according to RECIST v1.1 in RCC. Secondary endpoints included ORR in central nervous system hemangioblastomas, pancreatic neuroendocrine tumors (pNETs) as well as, duration of response (DOR), and treatment safety. The efficacy in retinal hemangioblastomas was assessed as an exploratory outcome.

### Study assessments

ORR was assessed per RECIST v1.1 by an independent radiologist, who retrospectively identified baseline lesions and evaluated follow-up imaging. Measurable target lesions were defined as solid lesions ≥10 mm in longest diameter. Up to five target and five non-target lesions were recorded per organ system. Best overall response during routine imaging follow-up was used for response assessment.

Renal solid tumors and complex cystic renal lesions with measurable solid components were considered target lesions. For CNS hemangioblastomas, solid components were assessed, with cystic portions measured when present. Measurable pancreatic neuroendocrine tumors were also included as target lesions.

Simple renal and pancreatic cysts, pancreatic cystadenomas, and other non-measurable lesions were classified as non-target lesions and assessed qualitatively. Unequivocal progression of non-target lesions constituted progressive disease.

Objective response rates were calculated separately for each organ. For rena cell carcinoma and pancreatic neuroendocrine tumors, the denominator included the number of patients with measurable target lesions per RECIST v1.1.

For CNS hemangioblastomas, the denominator included the number of all patients with CNS involvement, regardless of target-lesion status, because non-target lesions may be clinically significant in VHL disease. Complete response required complete disappearance of all CNS lesions, including non-target lesions, consistent with RECIST v1.1.

Retinal hemangioblastoma response was evaluated qualitatively based on ophthalmology documentation.

Patients were followed until the last available radiologic or clinical follow-up, with a data cutoff of February 2026.

### Statistical analysis

Descriptive statistics summarized baseline demographic and clinical characteristics. Categorical variables were reported as frequencies and percentages, and continuous variables as medians (range). Objective response rate was calculated with exact 95% confidence intervals (Clopper–Pearson method). DOR was defined from first documented response to disease progression or death and estimated using the Kaplan–Meier method.

Procedure rates per patient-year were calculated for the 3 years before and the follow-up period after belzutifan initiation. Rates were compared using a within-patient negative binomial regression model with an offset for person-time. Incidence rate ratios (IRRs) and 95% confidence intervals (CIs) were reported. The 3-year pretreatment window was selected because it provides a standardized assessment of procedure burden immediately preceding treatment, while minimizing the influence of procedures performed many years earlier.

Adverse events were graded per CTCAE v5.0. Analyses were performed using R version 4.3.0, and figures were generated using Python version 3.14.0.

## Results

### Patients’ population and baseline characteristics

A total of 199 patients treated with belzutifan were identified. Of these, 119 with sporadic RCC and 14 with other diagnoses (hemangioblastoma, *n* = 4; paraganglioma/pheochromocytoma, *n* = 6; SDHB-associated tumors, *n* = 3; ovarian cancer, *n* = 1) were excluded.

Sixty-six patients met criteria for VHL diagnosis, of whom 59 had baseline and follow-up imaging and were included.

Among 59 patients, the median age was 42 years (range, 17-82), and 59.3% were female. Median follow-up was 29 months (range, 3-49), and the within patients’ median time from VHL diagnosis to belzutifan initiation was 14 years (range, 1-47).

Prior to treatment, 27 patients (45.8%) underwent partial or complete nephrectomy, 34 (57.6%) CNS surgery, and 12 (20.3%) pancreas-related surgery. The median number of surgeries per patient was 2 (range, 1-3), 2 (range, 1-6), and 1 (range, 1-3), respectively (Table 1).

**Table 1.** Baseline characteristics of the cohort.

Characteristic	Patients (*n* = 59)
**Age, years—median (range)**	
** At study inclusion**	42 (17-82)
** At treatment start**	40 (16-81)
** At VHL diagnosis**	22.5 (4-59)
**Sex—no. (%)**	
** Female**	35 (59.3)
** Male**	24 (40.7)
**Race—no. (%)**	
** White**	57 (96.6)
** African American**	1 (1.7)
** Asian**	1 (1.7)
**ECOG performance status—no. (%)**	
** 0**	43 (72.8)
** 1**	6 (10.2)
** ≥2**	2 (3.4)
** Missing**	8 (13.6)
**VHL disease subtype—no. (%)** ^a^	
** Type 1**	44 (74.6)
** Type 2A**	8 (13.6)
** Type 2B**	7 (11.9)
** Type 2C**	0
**Baseline neoplasms—no. (%)**	
** Renal cell carcinoma**	29 (49.1)
** CNS hemangioblastoma**	53 (89.8)
** Pancreatic NET**	14 (23.7)
** Retinal hemangioblastoma**	13 (22.0)
**Previous surgery or ablative procedure—no. (%)**	
** Any prior procedure**	56 (94.9)
** Partial/radical nephrectomy**	27 (45.8)
** Kidney ablative procedures^b^**	19 (32.2)
** CNS surgery**	34 (57.6)
** CNS radiosurgery^c^**	14 (23.7)
** Pancreatic surgery^d^**	12 (20.3)
** Adrenalectomy^e^**	13 (22.0)
**No. of ablative/surgical procedures per patient—median (range)**	4 (1-24)

a Type 1 disease is associated with retinal and central nervous system hemangioblastomas, renal cell carcinoma, pancreatic cysts, and neuroendocrine tumors with a low risk of pheochromocytoma; type 2A with pheochromocytomas, retinal and CNS hemangioblastomas and a lower risk of renal cell carcinoma; type 2B with pheochromocytomas, renal cell carcinoma, and hemangioblastomas (retinal and CNS); and type 2C with pheochromocytomas only.

b Radiofrequency, cryoablation, and microwave ablation.

c Gamma Knife surgery.

d Includes total or partial pancreatectomy and tumor or cyst excision.

e Includes isolated adrenalectomy for pheochromocytoma and adrenalectomy performed with nephrectomy.

### Efficacy in renal cell carcinoma

Among the 59 patients, 29 (49.1%) had solid target RCC lesions, comprising a total of 60 target lesions with a median size of 2.1 cm (range, 1-15 cm). Of these 29, 18 patients had multiple target lesions (61.1%) and 11 had a single target lesions (37.9%). Four patients (6.8%) had metastatic RCC. Among the remaining 30 patients, 22 had non-target renal lesions only and were assessed qualitatively, while 8 had no renal lesions.

**Figure 1.**
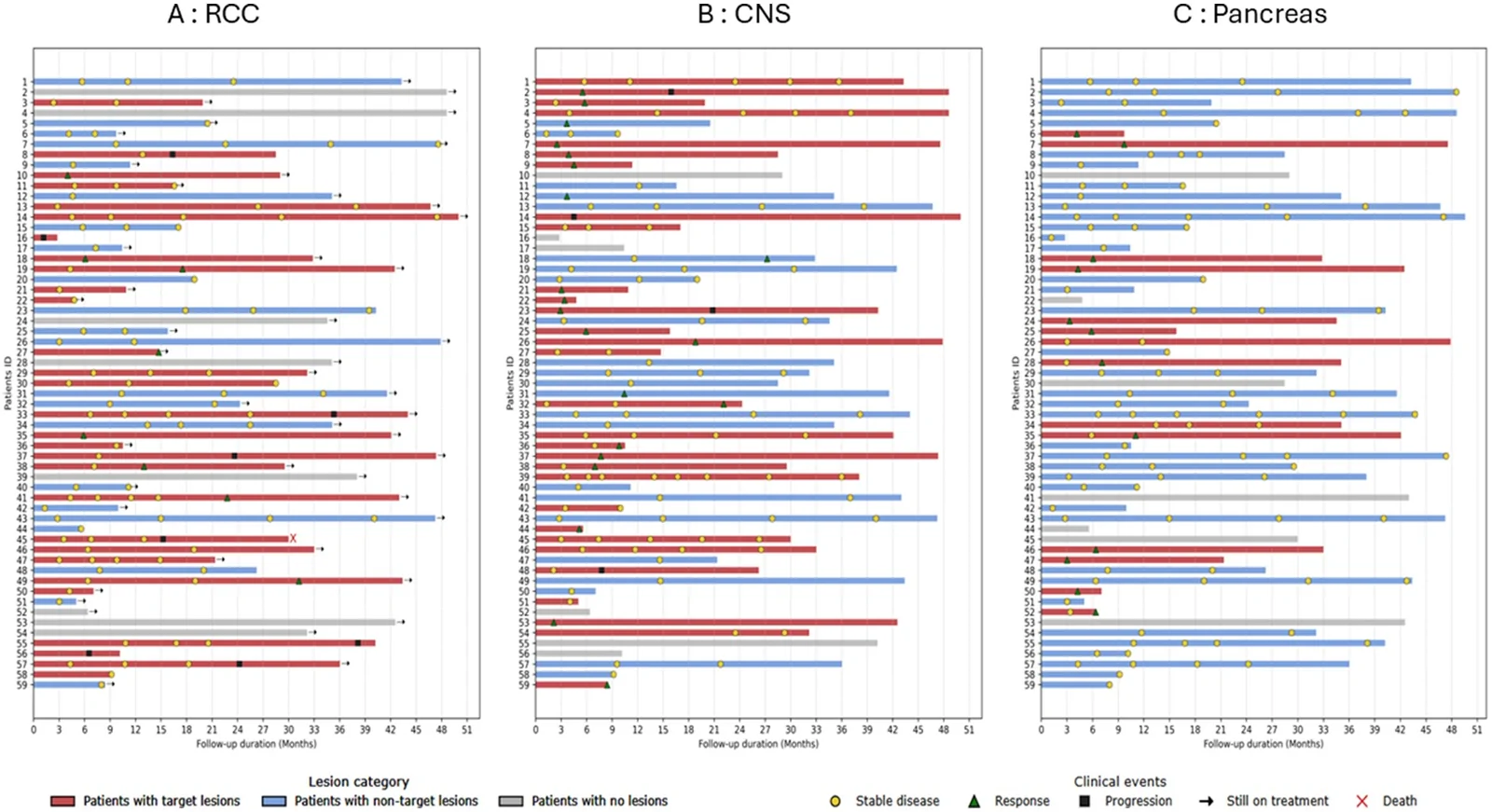
Swimmer plots showing follow-up duration and outcomes in (A) RCC, (B) CNS hemangioblastomas, and (C) pancreatic lesions. Each bar represents one patient (months from treatment start to last follow-up). Colors represent lesion category: target (red), non-target (blue), patients with no lesions in the corresponding organ (gray).

Confirmed response occurred in 8 patients (27.6%; 95% CI, 12.3-46.6), including 1 complete response. Median time to response was 13 months (range, 4-31), and median DOR was not reached (range, 0.0+ to 36+), with “+” indicating ongoing response. Stable disease was the best overall response in 13 patients (44.8%), 9 (31%) of whom had tumor shrinkage from baseline. Eight patients (27.6%) had confirmed progression. Patients with non-target lesions or no baseline renal disease remained progression-free (Figure 1A). Among patients with metastatic RCC at treatment initiation (*n* = 4, 6.8%), one had stable disease and three progressed (patients 16, 29, 45, and 56) (Figure 1A). Response or stable disease was observed in 83.3% (15/18) of patients with multiple target lesions versus 54.5% (6/11) in those with a single lesion.

Among patients with progression (*n* = 8, 27.6%), median time to progression was 19.3 months (range, 1-36). Five underwent tumor-reduction procedures, three of whom continued belzutifan beyond progression. Of the remaining three patients, two received cabozantinib plus nivolumab with clinical and radiographic improvement, while one with advanced metastatic RCC and multiple prior therapies was transitioned to hospice.

### Efficacy in CNS

CNS hemangioblastomas were present in 53 patients (89.8%), with objective response in 21 (39.6%; 95% CI, 26.5-53.9), including 3 complete responses. All complete responses occurred in patients with non-target lesions that fully resolved after treatment initiation. A total of 57 target lesions were identified (median size 1.4 cm, range 1-3.8), of which 30 originated in the cerebellum, 17 in the spine, and 10 in other locations (e.g., the brainstem). Median time to response was 5.5 months (range, 2-27), and median DOR was not reached (range, 0.0+ to 45.0+).

Progression occurred in 4 patients, including 2 with prior partial responses. Median time to progression was 12 months (range, 4-20). Following progression, 2 patients underwent tumor-reduction procedures; both discontinued treatment.

### Efficacy in pNET and retinal hemangioblastoma

Of 59 patients, 3 (5.1%) had prior total pancreatectomy and 52 (88.1%) had pancreatic lesions. Fourteen (23.7%) had measurable target pancreatic neuroendocrine tumors (pNETs), including 3 with biopsy-proven liver metastases (2 with prior total pancreatectomy; patients 24, 34, and 47; Figure 1C). A total of 17 target lesions were identified (median size 1.8 cm [range, 1-9.5]). Confirmed response occurred in 12 of 14 patients (85.7%; 95% CI, 57.2-98.2), including 2 complete responses and 2 partial responses in metastatic patients; the remaining 2 had stable disease. No patients experienced progression, and those with non-target lesions remained progression-free. Median time to response was 6 months (range, 3-11), and median DOR was not reached (range, 0.0+ to 38+).

Thirteen patients with retinal hemangioblastomas in 20 eyes were identified by chart review. Seventeen of 20 eyes (85%; 95% CI, 62.1-96.8) in 11 of 13 patients (84.6%) had improvement in retinal lesions based on the treating ophthalmologist’s assessment. One patient progressed after treatment interruption due to pregnancy and required multiple retinal procedures. Two patients had stable lesions. The median time to response in retinal lesions was 4 months (range, 2-15), and median DOR was not reached (range, 0.0+ to 37+ months).

### Tumor-reduction procedures

**Figure 2.**
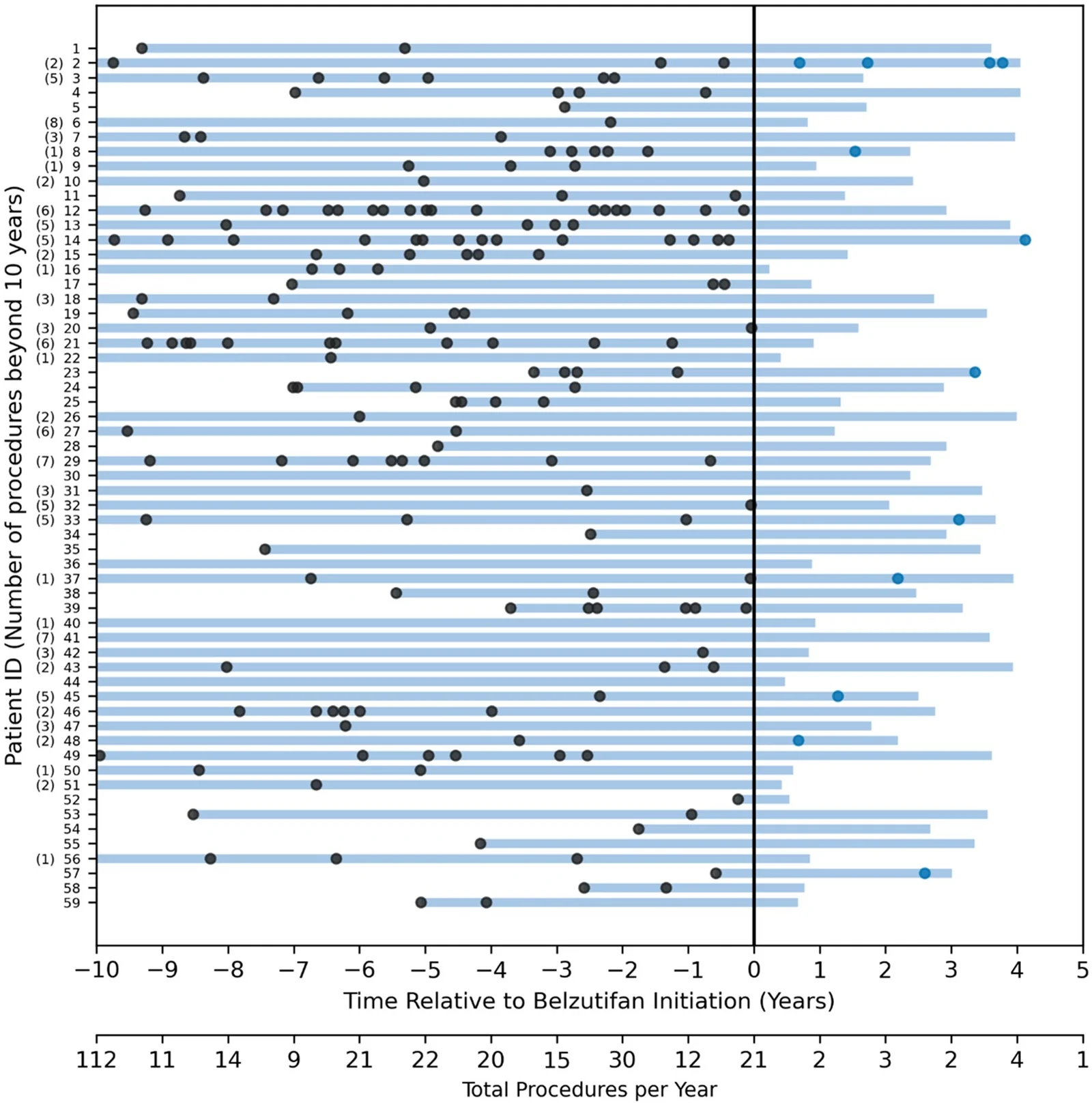
Procedures performed before and after belzutifan initiation (time 0). Each bar represents a patient. Black dots indicate pretreatment procedures, and blue dots post-treatment procedures. Numbers in parentheses denote the number of procedures performed beyond 10 years before treatment initiation for the corresponding patients. The leftmost value (112) represents the cumulative number of procedures performed more than 10 years before belzutifan initiation. Detailed breakdown of the curated procedures is provided in Table S1.

Over the 29 months of the median follow-up period, 9 patients (15.2%) underwent 12 tumor-reduction procedures after treatment initiation, including 4 retinal laser therapies in 1 patient, partial nephrectomy in 2 patients, renal ablation in 4 patients, cerebellar radiation in 1 patient, and surgical resection of a cerebellar lesion in 1 patient. In comparison, 281 procedures were performed before the treatment was started, of which 63 were performed in 33 patients (55.9%) during the 3 years before treatment. Additionally, the procedure rate decreased from 0.35 procedures per patient-year during the 3 years preceding treatment initiation to 0.08 procedures per patient-year during follow-up after treatment initiation. In a within-patient negative binomial regression analysis, this corresponded to a significantly lower procedure rate after treatment (IRR, 0.23; 95% CI, 0.12-0.47; *P *< .001; Figure 2).

### Safety and drug modifications

Fifty-eight patients (98.3%) experienced one or more adverse events (Table 2). The most common adverse event was anemia, which occurred in 51 patients (86.4%; Table S2). Treatment was interrupted in 24 patients (40.7%), with a median interruption duration of 0.9 month (range, 0.16-32); three of these patients subsequently discontinued treatment. Dose reductions occurred in 22 patients (37.2%).

**Table 2.** Adverse events of any cause in the cohort.

Adverse event	All patients (*n* = 59), no. (%)
**Any grade**	58 (98.3)
**Grade 3-5**	10 (16.9)
** Grade 3**	9 (15.2)
** Grade 4**	1 (1.7)
** Grade 5**	0
**Death**	
** Total^a^**	1 (1.7)
** Related to adverse events**	0

a One patient died due to complications of VHL disease.

At last follow-up, 31 patients (52.5%) remained on belzutifan 120 mg, 13 (22%) on 80 mg daily, and 3 (5.1%) on 40 mg daily. Twelve (20.3%) discontinued treatment: seven due to adverse events, three due to progression, one due to patient preference, and one due to logistics.

Among the 24 interruptions, adverse events were the most common reason in 19 (19/24; 79.1%) patients. Among these 19, the drug was successfully restarted in 17 (17/19; 89.5%) patients and discontinued in 2 (2/19; 10.5%). Other reasons for interruption were logistics in 3 patients (3/24; 12.5%) and pregnancy-related in 2 patients (2/24; 8.3%). Most interruptions (18/24; 75%) were ≤1 month; however, 6 patients (6/24; 25%) had interruptions for ≥5 months.

Most adverse events were grade 1-2 (Table S2). Grade ≥3 events occurred in 10 patients (16.9%), including one grade 4 status epilepticus, and treatment was resumed after 5 months.

Among patients with anemia, 3 (5.1%) required transfusions, 3 (5.1%) received erythropoiesis-stimulating agents (ESAs), and one patient (1.7%) had both. Two additional patients were advised against ESAs due to potential tumor-proliferative effects.

Grade 3 hypoxia occurred in 2 patients (3.4%), both of whom discontinued treatment.

## Discussion

In this multicenter retrospective study, we evaluated real-world outcomes of belzutifan across multiple organ systems in patients with VHL disease. To our knowledge, this represents the largest real-world cohort of belzutifan in this setting since its approval in August 2021.

We observed objective response rates (ORR) of 27.6% in RCC lesions, 39.6% in CNS lesions, and 85.7% in pancreatic neuroendocrine tumors (pNETs) over a median follow-up of 29 months. These findings are generally consistent with results reported in a subsequent analysis of the LITESPARK-004 trial with a similar duration of follow-up.^10^^,^^11^ However, the response rate among RCC lesions in our cohort was lower than that reported in the trial.^12^ This difference may be attributable to the smaller number of RCC patients in our cohort, whereas the presence of measurable RCC lesions was an inclusion criterion in LITESPARK-004. Patients with multiple target renal lesions demonstrated higher rates of stable or responsive disease compared with those with a single lesion. While the mechanism remains uncertain, this may reflect differences in tumor biology, including variable dependence on HIF-2α signaling. Additionally, the drug demonstrated comparable effectiveness to that in the trial in retinal lesions, with approximately 85% of patients having improvement based on ophthalmologic assessments.^13^ Our findings also confirm previously reported high response rates in pancreatic neuroendocrine tumors (pNETs),^14^ including two patients with liver metastases who achieved confirmed responses. Among responders, the median DOR was not reached, with only three progression events occurring in two patients, supporting the durability of response. Median time to response was shorter in pNET, retinal, and CNS hemangioblastoma compared to RCC, consistent with the findings from the LITESPARK-004.^7^^,^  ^13^ Previous studies have shown the real-world efficacy of belzutifan in patients with VHL disease.^15–17^ Similarly, Miller et al.^18^ demonstrated the efficacy of belzutifan using an external control arm compared with the single-arm LITESPARK-004 trial, consistent with our findings. Generally, the cohort reflects real-world heterogeneity, in which some patients receive belzutifan for lesions in certain organs while other are disease-free (Figure 1). In addition, the cohort included patients with heterogeneous disease severity, including patients with metastatic RCC, which may have contributed to the lower organ-specific response rates observed compared with LITESPARK-004.

Following treatment initiation, the overall rate of tumor-reduction procedures decreased from 0.35 to 0.08 per patient-year with IRR = 0.23, meaning that the procedure rate per patient-year after belzutifan was 23% of the procedure rate before belzutifan. These findings suggest that belzutifan may reduce the surgical burden in patients with VHL disease. However, although the within-patient analysis accounted for differences in person-time, longer follow-up is needed to determine whether procedures were avoided or delayed.

Treatment was well tolerated, with 47 (79.6%) patients remaining on therapy at last follow-up. Among the 19 patients who interrupted treatment due to AEs, the majority (17) restarted therapy. Importantly, several interruptions in our cohort were related to non-toxicity factors, including logistical issues and pregnancy-related holds.

Anemia was the most common AE; however, grade 3 anemia was uncommon and occurred in five patients. Hypoxia occurred in six patients, with grade 3 events reported in two. Notably, anemia and hypoxia events appeared less severe compared with reports in patients with sporadic RCC receiving belzutifan,^8^^,^^19^ possibly reflecting differences in baseline health and comorbidities between VHL and sporadic RCC populations.

### Limitations

This study has several limitations. Selecting a subgroup of patients with available radiologic imaging may have introduced selection bias; however, because the primary outcome was objective response rate according to RECIST v1.1, we aimed to ensure accurate assessments. All imaging assessments were performed by a single radiologist without independent adjudication, which may have introduced reader variability. Although data were curated by two reviewers, information bias cannot be excluded. AEs may be under-reported due to reliance on clinical notes. Because belzutifan was initiated during periods of active disease, the observed reduction in procedure rates may have been influenced by confounding by indication and regression to the mean, despite the within-patient analysis. Additionally, heterogeneity in imaging intervals and follow-up may have affected response or progression assessments.

## Conclusions

In this multi-center real-world cohort, belzutifan demonstrated activity across multiple von Hippel-Lindau disease–associated lesions, with durable responses and predominantly low-grade adverse events. Procedure rates were significantly lower following treatment, suggesting potential reduction in procedural burden. These findings complement clinical trials data and provide real-world evidence regarding the effectiveness of belzutifan in VHL disease.

## Supplementary Material

oyag331_Supplementary_Data

## Data Availability

The data supporting the findings of this study are available from the corresponding author upon reasonable request.
