# Supplementary material for: Clinical outcomes of HIF-2α inhibition in Von Hippel–Lindau disease: a multicenter real-world study
Source: Oncologist. 2026 Aug 18;31(10):oyag331. doi: 10.1093/oncolo/oyag331 (PMC13570587; doi:10.1093/oncolo/oyag331)
Supplement: Supplementary file 1 [file oyag331_supplementary_data.zip › Supplementary table 2.docx]

**Supplementary Table 2. Any-cause adverse events.**

| **Adverse Event** | **Any Grade**  **(n, %)** | **Grade (1–2)**  **(n, %)** | **Grade ≥3**  **(n, %)** |
| --- | --- | --- | --- |
| Anemia | 51 (86.4) | 46 (77.9) | 5 (8.5) |
| Fatigue | 36 (61.0) | 35 (59.3) | 1 (1.7) |
| Headache | 20 (33.9) | 20 (33.9) | 0 |
| Dizziness | 18 (30.5) | 18 (30.5) | 0 |
| Nausea | 13 (22.0) | 13 (22.0) | 0 |
| Abdominal pain | 11 (18.6) | 11 (18.6) | 0 |
| Constipation  Hypoxia | 10 (16.9)  8 (13.6) | 10 (16.9)  6 (10.2) | 0  2 (3.4) |
| Myalgia | 8 (13.6) | 8 (13.6) | 0 |
| Weight gain | 7 (11.9) | 7 (11.9) | 0 |
| LFTs increase | 7 (11.9) | 7 (11.9) | 0 |
| Muscle spasms | 6 (10.2) | 6 (10.2) | 0 |
| Blurry vision  Hypertension | 4 (6.8)  4 (6.8) | 4 (6.8)  3 (5.1) | 0  1 (1.7) |
| Thrombocytosis | 3 (5.1) | 3 (5.1) | 0 |
| Anxiety | 3 (5.1) | 3 (5.1) | 0 |
| Diarrhea | 3 (5.1) | 3 (5.1) | 0 |
| Creatinine increase | 2 (3.4) | 2 (3.4) | 0 |
| Peripheral edema | 2 (3.4) | 2 (3.4) | 0 |
| Arthralgia | 2 (3.4) | 2 (3.4) | 0 |
| Rash | 1 (1.7) | 1 (1.7) | 0 |
| Seizure | 1 (1.7) | 0 | 1 (1.7) |
