# Supplementary material for: Clinical outcomes of HIF-2α inhibition in Von Hippel–Lindau disease: a multicenter real-world study
Source: Oncologist. 2026 Aug 18;31(10):oyag331. doi: 10.1093/oncolo/oyag331 (PMC13570587; doi:10.1093/oncolo/oyag331)
Supplement: Supplementary file 1 [file oyag331_supplementary_data.zip › Supplementary table 1.docx]

**Supplementary Table 1. Breakdown of Tumor-Reduction Procedures by Organ System**

| **Organ System** |  | **Procedures Curated** |
| --- | --- | --- |
| Kidney |  | Partial nephrectomy; radical nephrectomy; and renal tumor excision |
|  |  | Radiofrequency ablation; cryoablation; and microwave ablation |
| Central Nervous System |  | Resection of CNS hemangioblastoma; craniotomy; laminectomy; and ventriculoperitoneal (VP) shunt placement. |
|  |  | Gamma Knife stereotactic radiosurgery. |
| Pancreas |  | Partial pancreatectomy; total pancreatectomy; and pancreatic tumor or cyst excision. |
| Adrenal |  | Isolated adrenalectomy and adrenalectomy performed with nephrectomy. |
| Retina |  | Laser photocoagulation; photodynamic therapy; cryotherapy; vitrectomy; and eye enucleation. |
